# Beyond Boundaries: A Comprehensive Review of Anthropometric Indices in Urban and Rural India

**DOI:** 10.7759/cureus.53944

**Published:** 2024-02-09

**Authors:** Astha Khurana, Amar Taksande, Revat J Meshram

**Affiliations:** 1 Pediatrics, Jawaharlal Nehru Medical College, Datta Meghe Institute of Higher Education and Research, Wardha, IND

**Keywords:** socio-economic factors, public health interventions, health disparities, urbanization trends, anthropometric indices

## Abstract

This comprehensive review examines anthropometric indices in the context of urban and rural India, shedding light on the dynamic interplay between lifestyle, socio-economic factors, and environmental influences on health outcomes. Analyzing indicators such as Body Mass Index (BMI), waist-to-hip ratio (WHR), and mid-upper arm circumference (MUAC), the study reveals distinct disparities between urban and rural populations. While rural areas face the challenges of undernutrition and stunting, urban environments grapple with the escalating prevalence of obesity and non-communicable diseases. The implications for public health underscore the need for tailored interventions, encompassing nutritional education, equitable healthcare access, and lifestyle interventions. The call-to-action advocates for collaborative efforts among policymakers, healthcare professionals, researchers, and communities to implement evidence-based strategies, advocate for policy reforms, and continually monitor anthropometric trends. This review serves as a roadmap for fostering healthier communities in India by addressing anthropometric disparities and steering toward a more equitable and sustainable future.

## Introduction and background

Anthropometric indices, encompassing measurements related to human physical dimensions and body composition, hold significant importance in understanding population health and well-being. These indices are valuable tools for assessing nutritional status, growth patterns, and overall health conditions. In the context of India, a nation characterized by diverse socioeconomic landscapes, anthropometric studies have played a crucial role in unraveling the complexities of health disparities [1].

Anthropometric indices provide quantifiable data that enable researchers and policymakers to evaluate populations' nutritional status and physical development. Height, weight, Body Mass Index (BMI), and other measurements offer insights into the prevalence of malnutrition, stunting, and obesity, among other health indicators. These metrics serve as critical markers for assessing the effectiveness of public health interventions and for devising targeted strategies to address health inequalities [1]. In the Indian context, where nutritional challenges persist alongside rapid urbanization and socioeconomic disparities, anthropometric measurements become essential for understanding health and development dynamics. By examining anthropometric indices, researchers can identify trends, disparities, and potential risk factors affecting diverse populations [2].

The history of anthropometric studies in India dates back several decades, reflecting a commitment to understanding and addressing health issues within the population. Early efforts were often driven by a desire to combat malnutrition and infectious diseases, focusing on child growth and development. Over time, these studies have evolved to encompass broader health concerns, including the impact of lifestyle changes, urbanization, and economic shifts on anthropometric measures [3]. The historical trajectory of anthropometric research in India mirrors the nation's journey through phases of agrarian economies, industrialization, and globalization. Such studies have contributed to formulating health policies and interventions to improve the population's overall well-being [3].

Against this backdrop, this comprehensive review aims to critically examine and synthesize existing literature on anthropometric indices, with a specific focus on urban and rural disparities in India. By delving into the country's historical context of anthropometric studies, we aim to shed light on the changing patterns of human development and health. Through a thorough analysis of urban and rural anthropometric studies, this review seeks to identify key trends, factors influencing stature disparities, and potential implications for public health policies. Furthermore, we aim to provide a nuanced understanding of how socioeconomic, cultural, and environmental factors contribute to variations in anthropometric measures across different regions in India.

## Review

Urbanization trends in India

Growth of Urban Population

India's urban population is undergoing substantial growth driven by natural increase, urban migration, and the reclassification of urban areas. Projections suggest that India will witness an addition of 416 million people to its urban population [4]. The urbanization rate in India saw a 1.5% increase in 2021, with an average annual percentage change indicating a 1.34% rise in the urban share of the population [5]. Over the past decade, there has been an almost 4% increase in urbanization, signaling a notable shift of individuals from rural to urban areas, often driven by employment opportunities [6]. In 2022, India's urban population exhibited a two percent growth rate [7]. According to the 2011 Census, the urban population in India surged to 377 million, with an annual growth rate of 2.76% from 2001 to 2011 [8]. These statistics underscore the persistent urbanization trends and the remarkable expansion of the urban population in India. The ongoing urbanization reflects dynamic demographic changes and the evolving socio-economic landscape in the country.

Lifestyle Changes and Health Implications

The impact of lifestyle changes on health in India has been profound, influenced by various factors, most notably the ramifications of the COVID-19 pandemic. This global health crisis has brought about significant lifestyle changes, affecting diet, physical activity, and mental stress levels [9]. Unhealthy dietary patterns, sedentary lifestyles, and tobacco consumption have emerged as prevalent contributors to the surge in lifestyle diseases in India, including cardiovascular diseases, obesity, and metabolic disorders [10,11]. The nationwide lockdown implemented during the pandemic further exacerbated these challenges, leading to substantial shifts in diet, sleep, and personal habits among a large population segment [12].

These observations underscore the imperative for continuously monitoring lifestyle behaviors and emphasize promoting healthy dietary habits, physical activity, and mental well-being. Such efforts are paramount to mitigating the impact of lifestyle-related health issues in India. As the nation grapples with evolving health challenges, proactive measures to foster positive lifestyle changes can play a pivotal role in averting and managing the adverse health effects of contemporary lifestyle trends.

Socioeconomic Factors Affecting Urban Anthropometry

Urbanization trends in India are intricately intertwined with profound socioeconomic factors, exerting a substantial influence on urban anthropometry. The swift urbanization in the country has given rise to escalating socioeconomic disparities and the emergence of health inequalities among urban children [13]. Projections indicate that India's urban population is poised to reach 900 million by 2050, with nearly half of the populace residing in urban areas [13]. This urbanization wave profoundly affects the population's health, nutrition, and anthropometric measures.

Studies affirm that urbanization and associated socioeconomic factors leave a discernible imprint on anthropometric indices in India. For instance, research comparing anthropometric assessments across the rural-urban interface of Bangalore revealed a higher prevalence of underweight among women (13.56%) compared to men (8.88%) [14]. Another study, focusing on the urban-rural comparison of anthropometry and menarcheal status among adolescent school-going girls in Jodhpur, Rajasthan, India, found that the anthropometric measurements exceeded the NCHS reference standard and Zanvar et al. for that age group [15].

The double burden of malnutrition, encompassing both undernutrition and overnutrition, has become a growing concern in India. A study examining trends and inequalities in undernutrition and overnutrition identified an increase in the prevalence of stunting among children and underweight among adults in slum areas between 2006 and 2016 [16]. This underscores how rapid urbanization and associated socioeconomic factors have contributed to the dual challenges of malnutrition in India.

Comparative analysis: urban versus rural anthropometric trends

BMI Trends

The urban-rural disparity in BMI has been a focal point of investigation in various regions, notably China and Montenegro. A Chinese study revealed that urban adults tended to exhibit higher BMI than their rural counterparts, with the overall urban-rural BMI disparity diminishing since 2004, particularly among women [17]. Similarly, a study in Montenegro found that girls and boys in urban areas displayed lower BMI values than their rural counterparts, showcasing significant differences in BMI between urban and rural adolescents [18]. These findings underscore the BMI variations in urban and rural populations across diverse regions.

In Bengaluru, India, a study compared anthropometric assessments along the rural-urban interface, discovering a higher prevalence of underweight among women than men, with occupational differences noted across the rural-urban gradient [14]. Additionally, a Chinese study noted a substantial BMI increase among urban and rural participants over the past 18 years. Before 2006, urban participants exhibited higher BMI and waist circumference than their rural counterparts. However, this urban-rural gap became statistically insignificant in 2009 and 2011 [19].

Furthermore, a global study published in *Nature* highlighted that the escalating rural body mass index serves as a primary driver of the global obesity epidemic in adults [20]. This underscores the pivotal role of urbanization as a contributing factor to the worldwide obesity epidemic [20]. These collective studies offer valuable insights into the urban-rural differences in BMI and shed light on the influence of urbanization on trends in body mass index.

Waist-to-Hip Ratio Variations

The waist-to-hip ratio (WHR), a measure of body fat distribution, is calculated by dividing the waist circumference by the hip circumference. In the Indian context, numerous studies have delved into anthropometric measures, particularly WHR, and their correlation with non-communicable diseases (NCDs) across various population groups. An Indian study scrutinized physical body indices, including WHR, among individuals aged 45 and above, aiming to unravel associations with different non-communicable morbidities [21]. This research yielded valuable insights into the connection between WHR and NCDs among the elderly in India [21]. Another study compared the relative waist circumference between Asian Indian and US adults, revealing that Asian Indian women exhibited a higher mean WHR than women from other ethnic groups. This accentuates the diversity in body fat distribution among distinct populations [22].

A rural-urban comparison in North India explored differences in BMI and WHRs between rural and urban populations, shedding light on anthropometric measure variations across diverse settings [23]. Moreover, studies have indicated that waist circumference correlates more effectively with body mass index than WHR in Asian Indians. This underscores the importance of considering multiple anthropometric measures when assessing abdominal obesity in this population [24]. Collectively, these studies enhance our understanding of WHR variations and their implications for health outcomes in various demographic groups in India. They underscore the significance of incorporating WHR into discussions around NCDs and body fat distribution among diverse populations.

Gender and Age Disparities

Disparities in anthropometric indices related to gender and age have garnered attention in various studies. Search results underscore distinctions in anthropometric measures between men and women, shedding light on how age influences these metrics. A study in China revealed that, particularly in rural boys, anthropometric indices might be more suitable for screening dyslipidemia than for girls [25]. The research assessed how anthropometric indices predicted dyslipidemia based on gender and living areas in Chinese primary school children. Research in low- and middle-income countries demonstrated that urban children tend to be taller and heavier than their rural counterparts, with this urban-rural gap being more notable in boys [26]. This suggests potential gender-specific differences in growth patterns between urban and rural populations.

An investigation into older adults in India evaluated physical body indices such as BMI, waist circumference (WC), and WHR among men and women aged 45 and above, aiming to explore associations with various non-communicable morbidities [21]. Another study comparing health status measurements of older women in urban and rural communities found that rural women exhibited a higher BMI [27]. Collectively, these findings emphasize the existence of gender and age disparities in anthropometric indices, carrying implications for public health interventions and policies. A nuanced understanding of these differences is crucial for devising targeted strategies that address the distinctive health needs of men, women, and diverse age groups.

Health implications

Urban Health Challenges

Obesity and non-communicable diseases: Urbanization in India has brought forth a spectrum of health challenges, prominently marked by the rise in obesity and NCDs. Research indicates that urban populations exhibit a higher prevalence of NCD risk factors, including physical inactivity, overweight, and obesity, compared to their rural counterparts [28]. The urban environment, characterized by high-volume traffic, compromised air quality, and a shortage of safe public spaces and recreation/sports facilities, can further impede physical activity [29]. Moreover, urban living introduces new hazards and concentrates health risks, disproportionately affecting the poorest and most vulnerable segments of society, thereby exacerbating health inequities [30]. The prevalence of obesity and overweight is notably higher in urban areas compared to rural regions, a trend expected to intensify with the ongoing rapid urbanization of India [31]. A significant shift in the disease spectrum from communicable to non-communicable diseases, particularly cardiovascular diseases, diabetes mellitus, and stroke, poses a substantial public health challenge in India [28]. Consequently, addressing these health challenges requires comprehensive interventions and approaches to prevent risk factors. This includes measures such as regulating unhealthy foods, urban planning initiatives to promote physical activity, and fostering partnerships with stakeholders in industry, labor, education, and urban planning [28]. A multi-sectoral approach is imperative to effectively confront the health challenges posed by the urban environment in India. This involves coordinated efforts in urban planning, targeted public health interventions, and strategic partnerships with diverse stakeholders. By addressing the root causes and risk factors collaboratively, India can work towards creating healthier urban environments and mitigating the growing burden of obesity and NCDs.

Sedentary lifestyles: Sedentary lifestyles pose a substantial health challenge in urban areas, exacerbated by the impact of urbanization on job structures and the resultant decrease in both occupational and leisure-time physical activity [32]. Numerous urban factors further hinder physical activity, including high-volume traffic, extensive reliance on motorized transportation compromised air quality, and a shortage of safe public spaces and recreation/sports facilities [29]. The consequences of sedentary lifestyles are far-reaching, contributing to heightened all-cause mortality, increased cardiovascular disease mortality, elevated cancer risks, susceptibility to obesity, and a higher likelihood of developing type 2 diabetes [33]. Notably, the mean daily duration of sedentary behavior is 8.3 hours among the Korean population and 7.7 hours globally [33]. The scarcity of available spaces for exercise, such as parks, pedestrian walkways, and sports or leisure facilities, emerges as a significant factor fostering sedentary lifestyles [33]. Effectively addressing the challenges of sedentary lifestyles in urban areas necessitates a comprehensive, multi-faceted approach. This approach should encompass the creation of safe and accessible public spaces, the promotion of active transportation alternatives, and the encouragement of physical activity through community-based programs and policies. By addressing the environmental barriers that hinder physical activity, urban areas can foster a culture of health and well-being, ultimately mitigating the adverse effects of sedentary lifestyles.

Rural Health Challenges

Undernutrition and stunting: The prevalence of undernutrition, specifically stunting, among children in India stands as a significant and pressing public health concern, impacting both urban and rural areas. Numerous studies have shed light on the substantial burden of undernutrition, focusing on critical indicators such as stunting, wasting, and underweight. A study conducted in Maharashtra, India, illuminated the high prevalence of stunting and underweight among children in rural and urban slum areas, emphasizing the urgent necessity for comprehensive assessments of children's nutritional status to inform health policies [34]. Further insight into the localized disparities of stunting prevalence was provided by a study that mapped childhood undernutrition at the village level in India. This research demonstrated stark variations in stunting rates, surpassing 70% in certain villages, underscoring the importance of understanding and addressing these disparities on a local level [35]. Additionally, research in Telangana and Andhra Pradesh, encompassing both urban and rural areas, aimed to grasp the burden of malnutrition across different age groups [36]. This study emphasized the adverse health impacts and social consequences associated with under- and over-nutrition, highlighting the nuanced challenges diverse populations face [36]. The persistent nature of undernutrition, especially stunting, in both urban and rural Indian settings is evident from these studies. They stress the critical requirement for targeted interventions and strategies to alleviate the substantial burden of undernutrition among children, considering the local disparities and demographic dynamics at play. The identified factors contributing to undernutrition encompass poverty, insufficient food intake, inappropriate dietary practices, and limited access to proper medical care. Effectively addressing these challenges necessitates a comprehensive, multi-sectoral approach that involves collaboration between the government, private sector, and civil society. Such concerted efforts are crucial for accelerating progress toward achieving the Sustainable Development Goals (SDGs) related to nutrition and child health [37].

Occupational health risks: Occupational health risks pose a significant and complex challenge in rural areas, impacting diverse professions such as farming, agriculture, mining, and logging. Individuals in these occupations, including farmers and agricultural workers, are exposed to a spectrum of risks ranging from physical injuries to behavioral and mental health issues, as well as chronic health problems like vascular disorders and malignancies [38]. Agriculture, in particular, stands out as one of the most hazardous industries in the United States, where transportation incidents, notably tractor rollovers, rank as the leading cause of fatalities [39]. Farm workers contend with various health concerns, including psychological illness, injuries, parasites, skin diseases, and the potential hazards associated with agrichemicals [38]. Moreover, farm owners face the added risks of stress and exhibit notably high rates of suicide [38]. In addition to occupational hazards, rural residents may encounter challenges related to limited access to healthcare services, contributing to suboptimal health outcomes [40,41]. Statistics reveal that rural Americans are more prone to premature mortality from leading causes such as heart disease, cancer, and lung diseases [42]. Furthermore, they exhibit higher rates of cigarette smoking, high blood pressure, and obesity, coupled with lower levels of leisure-time physical activity and reduced seatbelt usage compared to their urban counterparts [40]. The multifaceted health challenges in rural areas underscore the imperative for heightened attention and dedicated resources. Efforts should focus on improving health outcomes by implementing enhanced public health programs that promote healthier behaviors and ensure better access to healthcare services [40]. This comprehensive approach is essential for addressing the unique occupational health risks and broader health disparities rural communities face.

Methodological approaches in anthropometric studies

Anthropometric studies employ diverse methodological approaches to measure and evaluate various body parameters. These methodologies encompass traditional manual measurements, advanced 3D body scans, and dynamic anthropometry. Key anthropometric measurements, including height, weight, BMI, waist circumference WC, and WHR, serve as critical indicators for assessing nutritional status, body composition, and health risks associated with NCDs across distinct population groups. The Centers for Disease Control and Prevention (CDC) underscores the significance of anthropometry in appraising nutritional status, growth, and health, particularly in children and adults [1]. In addition, 3D body scanners have emerged as valuable tools for conducting detailed anthropometric measurements, providing comprehensive insights into body size and shape [43]. Moreover, a study has demonstrated the feasibility of conducting anthropometric assessments via videoconference, presenting a cost-effective and valid alternative for collecting body composition measurements, especially in geographically dispersed samples [44]. These methodological approaches play a pivotal role in comprehending diverse populations' physical characteristics and health implications, contributing significantly to assessing nutritional status and disease risk and designing tailored interventions and products.

Policy implications

Healthcare Planning and Resource Allocation

Healthcare planning and resource allocation: Anthropometric indices play a pivotal role in assessing the health status of individuals and populations, providing valuable insights for healthcare planning and resource allocation. Studies have demonstrated the influence of socio-demographic characteristics, such as age, gender, and education level, on health status, allowing for the customization of healthcare services and interventions based on specific demographic groups. This tailored approach ensures that resources are allocated efficiently, addressing different populations' unique needs and challenges and promoting more effective healthcare outcomes [45].

Disease management: Anthropometric indices contribute essential information for establishing comprehensive strategies to improve disease management. Research confirming the impact of dietary habits on health status, as revealed through anthropometric studies, enables the design of targeted interventions to promote healthier eating habits and reduce the risk of chronic diseases. By incorporating anthropometric data into disease management initiatives, healthcare professionals can develop more precise and effective interventions that address the root causes of health issues and enhance overall well-being [45].

Nutritional assessment: Anthropometric data serve as valuable tools for assessing the nutritional status of individuals and populations. The long-term tracking of growth and weight trends in the U.S. population using National Health and Nutrition Examination Survey (NHANES) anthropometry data exemplifies the importance of this information in understanding health and dietary status, disease risk, and body composition changes over time. Nutritional assessment based on anthropometric indices provides critical data for developing targeted interventions, dietary recommendations, and public health initiatives to improve overall nutritional health [46].

Monitoring and surveillance: Anthropometric indices are instrumental in monitoring and surveilling changes in the nutritional status of populations. Systematic literature reviews comparing the performance of different anthropometric indices/measures for detecting changes over short and long terms provide a robust foundation for identifying trends. This information is crucial for policymakers, enabling them to make informed decisions to address nutritional issues and allocate resources effectively. Anthropometric monitoring and surveillance offer a dynamic and responsive approach to public health challenges, facilitating timely interventions and policy adjustments [47].

Impact on healthcare resources: Anthropometric indices, particularly in assessing obesity, directly impact healthcare resources. Studies have shown a positive association between obesity rates and healthcare costs, as well as the length of stay for patients. This underscores the importance of considering anthropometric data, such as obesity rates, in healthcare planning and resource allocation. The insights derived from these indices inform strategic decisions on resource distribution, ensuring that healthcare services are adequately equipped to address the challenges posed by varying health statuses, including obesity-related conditions [48].

Public Health Interventions

Physical activity interventions: The systematic review and meta-analysis protocol designed to evaluate the effects of physical activity interventions on anthropometric indicators and health indices in Chilean children and adolescents represent a significant contribution to the field [49]. This study aims to provide a standardized methodology for systematically reviewing existing evidence and synthesizing the most widely used field-based methods and health indices in assessing the impact of physical activity interventions on anthropometry. By employing rigorous review protocols, this research seeks to offer valuable insights into the effectiveness of physical activity interventions, providing evidence-based information that can inform future public health initiatives to improve Chilean youth's health and well-being.

Anthropometric, biochemical, socio-demographic, and dietary factors: The study investigating the impacts of anthropometric, biochemical, socio-demographic, and dietary factors on the health status of urban corporate individuals in a developing country sheds light on the complex interplay of various influences on health outcomes [45]. The findings underscore the significant role of socio-demographic characteristics, including age, gender, and education level, in determining health status. Moreover, the research emphasizes the importance of considering dietary habits, such as sugar-containing drinks and fast food, particularly in urban areas. This comprehensive approach to understanding health determinants highlights the need for targeted interventions that address multiple factors to improve overall health and mitigate health disparities in urban corporate populations.

Obesity and healthcare resources: The study exploring the impact of obesity on healthcare resources provides crucial insights into the association between body mass and healthcare costs and length of stay for patients [48]. This research underscores the importance of considering obesity rates and anthropometric data in healthcare resource planning. The positive association between increasing body mass and healthcare utilization necessitates strategic planning and allocation of resources to accommodate the healthcare needs of individuals with higher body mass. Public health interventions informed by these findings can target various factors, including physical activity, dietary habits, and socio-demographic characteristics, to improve health outcomes and reduce health inequalities. Incorporating systematic reviews and standardized methodologies enhances the robustness and reliability of such interventions, providing policymakers and healthcare providers with evidence-based tools for decision-making.

Education and Awareness Programs

Youth for Human Rights: Youth for Human Rights is a commendable organization dedicated to empowering young people and fostering groups that promote human rights. Their multifaceted approach encompasses educational programs, contests, projects, awareness campaigns, and outreach programs. Through art, essays, poetry contests, and mural projects, they engage creatively with youth, encouraging expression and understanding of human rights issues. Additionally, youth summits and human rights awareness campaigns amplify the impact of their educational initiatives. The inclusion of a human rights curriculum underscores their commitment to instilling a sense of responsibility and awareness among young minds, cultivating a generation that actively advocates for and safeguards human rights [50].

Mental health education and awareness programs: Mental health education and awareness programs play a crucial role in dismantling stigmas surrounding mental health conditions. These programs offer a wealth of information and resources, covering topics such as mental health conditions, available treatments, and local resources. By providing educational materials on recognizing signs of individuals in need, these initiatives contribute to a more informed and supportive community. The focus on awareness aims to destigmatize mental health, encourage open conversations, and foster an environment where seeking help is normalized, ultimately promoting mental well-being [51].

National Heart, Lung, and Blood Institute (NHLBI) education and awareness programs: The NHLBI's commitment to public health shines through its education and awareness programs. Targeting heart, lung, blood, and sleep diseases and disorders, these initiatives strive for positive change on a national scale. By developing comprehensive programs, the NHLBI aims to lower the risk factors associated with these health issues. This multifaceted approach involves educating the public, fostering awareness, and advocating for lifestyle changes that can improve cardiovascular and respiratory health, enhancing overall public health [52].

Early awareness programs: Early awareness programs address barriers that hinder low-income students from pursuing higher education. By identifying and mitigating these barriers, such programs pave the way for academic success. Offering scholarships as incentives motivates students and is a tangible means of support. These programs recognize the importance of early intervention, aiming to eliminate obstacles and empower students to achieve their educational goals, thereby promoting equality of educational opportunities [53].

Project AWARE: Project AWARE (Advancing Wellness and Resiliency in Education) stands out as a program with a comprehensive focus on developing a sustainable infrastructure for school-based mental health and substance abuse services. Prioritizing school climate improvement and trauma-informed programming, Project AWARE addresses the holistic well-being of students. By integrating mental health and substance abuse support into educational settings, the program acknowledges the interconnectedness of mental health and academic success, contributing to a healthier and more supportive learning environment [54].

Future directions for research

Emerging Anthropometric Indices

ABSI: The ABSI (A Body Shape Index) represents a contemporary alternative to traditional BMI measurements. This index considers not only the overall weight but also the distribution of fat and muscle mass. By incorporating a more nuanced understanding of body composition, ABSI offers a refined metric for assessing health risks associated with obesity and related conditions [55].

BRI: The BRI (Body Roundness Index) is a valuable metric for gauging central obesity. It is derived from the waist circumference to hip circumference ratio, providing a more comprehensive measure of fat distribution. BRI contributes to a more accurate assessment of obesity-related health risks by focusing on central adiposity, highlighting its significance in health screenings and interventions [55].

Waist-to-height ratio: The waist-to-height ratio (WtHR) is calculated by dividing waist circumference by height, offering a straightforward yet effective tool to assess the risk of obesity-related health issues. This index considers the proportion of abdominal fat relative to overall height, providing a more personalized indicator of health risks associated with central adiposity [56].

Conicity Index (CI): The Conicity Index (CI) adds a dimensional aspect to assessing metabolic syndrome risk. Based on the ratio of waist circumference to the square of height, CI offers insights into the distribution of body fat and its implications for metabolic health. This index is particularly valuable in identifying individuals at risk of metabolic disorders and guiding preventive measures [56].

These emerging anthropometric indices have been increasingly used in studies to investigate their association with various health conditions, including hypertension and other NCDs [55,56]. However, there is still limited research on these indices in the Indian population [55]. Further studies on these emerging anthropometric indices in different demographic groups and regions in India can provide valuable insights into their association with health outcomes and help inform targeted interventions for preventive and treatment measures.

Longitudinal Studies and Tracking Changes

Quality of anthropometric data in India's National Family Health Survey: This study employs a cross-classified multilevel model to meticulously analyze the factors influencing the quality of child anthropometric data within India's National Family Health Survey (NFHS) framework [57]. By disentangling the confounding effects of regions/districts and field teams, the research reveals a positive trajectory in data quality over time. However, it emphasizes the critical need to assess the reasons for data variance, highlighting the importance of further developing tools and techniques to enhance anthropometric measures' precision and reliability. This study contributes significantly to the ongoing efforts to refine data collection methodologies in large-scale health surveys like the NFHS.

Comparison of anthropometry measures between urban and rural aging cohorts in India: Utilizing data from the Tata Longitudinal Study, this research endeavors to observe and understand variations in various anthropometric measurements among aging participants in both urban and rural settings in India [58]. By shedding light on the differences in anthropometric measures between these two distinct cohorts, the study emphasizes the importance of tailoring interventions and policies based on urban and rural demographics. The findings provide valuable insights to guide targeted health initiatives, addressing the specific needs and challenges aging populations face in different environments.

An assessment of anthropometric indices and its association with NCDs among the older adults of India: Drawing on secondary data from the Longitudinal Ageing Survey's first wave in India, this study focuses on evaluating the physical body indices, including BMI, WC, and WHR, among older adults aged 45 and above [21]. The research seeks to uncover associations between these anthropometric indices and various non-communicable morbidities, offering critical insights into the prevalence of NCDs among the elderly population in India. This study underscores the potential of longitudinal research in comprehending changes in anthropometric indices and their correlation with health outcomes. The findings pave the way for informed interventions and policies to improve health outcomes and reduce health disparities, especially among older adults in India. Further research in this area holds promise for refining strategies addressing the unique health challenges diverse demographic groups face.

Technological Advances in Anthropometric Measurement

Noncontact anthropometric measurement: The adoption of noncontact methods, exemplified by Infrared-Ultra Wideband (IR-UWB) radar, represents a noteworthy advancement in anthropometric assessment. In a preclinical trial, the application of this technology demonstrated promise, particularly in scenarios involving young, uncooperative infants or children. By eliminating the need for physical contact, noncontact anthropometric measurement methods like IR-UWB radar provide a practical solution for challenging demographic groups and mitigate the risk of infectious disease transmission, making them particularly relevant in healthcare settings [59].

Remote and parent-reported measurements: The paradigm shift towards remote anthropometry introduces innovative approaches to gathering anthropometric data in pediatric populations. This methodological evolution enables the integration of parent-reported measurements into epidemiological models, offering a valuable tool for identifying associations between health outcomes and growth patterns. The rise of technology facilitates the seamless collection of data, providing a more comprehensive understanding of pediatric anthropometry and its implications for health and development [60].

Advancements in equipment and techniques: The continuous evolution of anthropometric research is accompanied by advancements in equipment and measurement techniques. Utilizing calibrated weight scales, stadiometers, knee calipers, skinfold calipers, and non-stretchable tape measures contributes significantly to enhancing the accuracy and reliability of anthropometric measurements. By employing state-of-the-art tools and techniques, researchers can minimize measurement errors and ensure that the collected data reflects precise body composition and growth indicators. This emphasis on technological sophistication underscores the commitment to advancing the quality and precision of anthropometric assessments [1].

Applications in adult populations: Anthropometric measurements play a crucial role in assessing adults' health and dietary status, offering valuable insights into future disease risk. In this context, these measurements serve as essential tools for health practitioners to monitor and evaluate the well-being of individuals, providing indicators that aid in early detection and prevention of potential health issues. Furthermore, anthropometric assessments contribute to optimizing competitive performance in athletes by determining body composition. This application extends to identifying conditions such as eating disorders, highlighting anthropometric measurements' versatility in addressing health and performance-related concerns among adults [1].

Epidemiological insights: Anthropometric indices, including widely utilized metrics like BMI and WHR, are integral to epidemiological research. These indices are extensively studied in longitudinal studies, functioning as measures of underweight, overweight, and obesity. Additionally, they serve as predictors of various health outcomes, providing valuable data for researchers to explore correlations between anthropometric measures and overall health. As future directions in anthropometric measurement, there is a concerted effort to enhance the accuracy, reliability, and accessibility of these assessments. This emphasis aims to make anthropometric data more helpful in understanding growth, nutrition, and health in diverse populations, ultimately contributing to more effective public health interventions and health promotion strategies [61]

## Conclusions

In conclusion, the comprehensive review of anthropometric indices in urban and rural India illuminates the intricate interplay of lifestyle, socio-economic factors, and environmental influences on health outcomes. The recapitulation of findings underscores the stark contrasts in Body Mass Index (BMI), waist-to-hip ratio (WHR), and mid-upper arm circumference (MUAC) between urban and rural settings, revealing the prevalence of undernutrition and stunting in rural areas alongside the escalating challenges of obesity and non-communicable diseases in urban environments. The implications for public health are clear, necessitating targeted interventions that address the unique needs of diverse populations. The identified disparities underscore the importance of nutritional education, equitable healthcare access, and lifestyle interventions to foster healthier communities. The call to action urges policymakers, healthcare professionals, researchers, and communities to collaborate to implement evidence-based strategies, advocate for policy reforms, and continuously monitor anthropometric trends. By embracing a holistic approach and prioritizing the well-being of individuals across different contexts, India can aspire to bridge anthropometric disparities and pave the way for a healthier and more equitable future.
